# In-hospital complications and treatment decision-making in osteoporotic pelvic ring fractures: The Role of the OF-Pelvis Score

**DOI:** 10.1016/j.bas.2026.107481

**Published:** 2026-10-27

**Authors:** Felix C. Kohler, Philipp Schenk, Pamela Schanderl, Volker Zimmermann, Bernhard W. Ullrich, Martin Naisan, Erik Wegner, Michael Müller, Frank Hartmann, Klaus J. Schnake, Imke Schmerwitz, Lars Behr, Max J. Scheyerer, Robert Pätzold, Sebastian Grüninger, Georg Osterhoff, Ulrich J.A. Spiegl

**Affiliations:** a Department of Trauma, Hand and Reconstructive Surgery and Orthopedics, Universitätsklinikum Jena – Friedrich-Schiller-University, Jena, Germany; b Innovationhub for Musculoskeletal Surgery Halle (IMCH), BG Klinikum Bergmannstrost Halle gGmbH, Halle (Saale), Germany; c Department of Orthopaedics, Trauma and Plastic Surgery, University Hospital Leipzig, Leipzig, Germany; d Department of Trauma and Orthopedic Surgery, Klinikum Traunstein, Traunstein, Germany; e Department of Trauma and Reconstructive Surgery, BG Klinikum Bergmannstrost Halle, Halle, Germany; f Spine Center, St. Josef-Hospital Wiesbaden, Wiesbaden, Germany; g Department of Trauma and Orthopedic Surgery, Universitätsmedizin Mainz – Johannes Gutenberg-University, Mainz, Germany; h Department of Orthopedic and Trauma Surgery, Universitätsklinikum Schleswig-Holstein, Campus Kiel, Germany; i Department of Trauma Surgery, Diakonie Krankenhaus, Bad Kreuznach, Germany; j Center for Spinal and Scoliosis Surgery, Malteser Waldkrankenhaus St. Marien, Erlangen, Germany; k Clinic for Orthopedic Surgery, Sports Traumatology and Trauma Surgery, Städtisches Klinkum Wolfenbüttel, Wolfenbüttel, Germany; l Interdisciplinary Center for Spine and Neurotrauma, Sana Klinikum Borna, Borna, Germany; m Department of Trauma and Orthopedic Surgery, Universitätsklinikum Düsseldorf – Heinrich Heine University, Düsseldorf, Germany; n Department of Trauma and Reconstructive Surgery, BG Unfallklinik Murnau, Murnau, Germany; o Department of Orthopedics and Traumatology, Klinikum Nürnberg, Nuremberg, Germany; p Clinic for Trauma Surgery and Orthopaedics, Munich Harlaching, Sanatoriumspl. 2, 81545 München, Germany; q Clinic for Trauma- and Reconstructive Surgery UMH, Halle, Germany

**Keywords:** Osteoporotic pelvic fractures, Pelvic ring fractures, OF-Pelvis score, Complications, Geriatric trauma, Frailty

## Abstract

**Introduction:**

Osteoporotic pelvic ring fractures (OPRFs) are associated with immobility-related complications and functional decline. The OF-Pelvis classification and score support treatment decision-making, but their relationship with in-hospital complications remains unclear.

**Research question:**

This study investigated whether the OF-Pelvis Score, fracture morphology, treatment modality, or patient-related factors predict in-hospital complications and short-term functional recovery.

**Material and methods:**

In this prospective multicentre cohort study, 390 patients with OPRFs treated at 14 German trauma centers were analysed. Fractures were classified according to the OF-Pelvis system, and score-based treatment recommendations were compared with actual treatment. Predictors of complications were assessed by logistic regression, and functional outcomes from treatment decision to discharge were analysed using repeated-measures general linear models.

**Results:**

Treatment information was available for 386 patients; 254 (66%) underwent surgery and 132 (34%) received conservative treatment. Complications occurred in 102 patients (26%). Treatment was concordant with the OF-Pelvis Score recommendation in 90% of cases, supporting its role in treatment decision-making. However, the score did not predict in-hospital complication risk. Age was the only independent predictor of complications, whereas treatment modality, baseline mobility, pain, and ASA classification were not independently associated with adverse events. Functional outcomes improved significantly during hospitalization.

**Discussion and conclusion:**

The OF-Pelvis Score reflected real-world treatment decisions but did not predict complication risk. Complications were primarily associated with patient-related factors, particularly age, supporting targeted geriatric co-management and complication prevention.

## Highlights

• In-hospital complications occurred in 26% of patients with OPRFs.
• Age was the only independent predictor of in-hospital complications.
• The OF-Pelvis Score showed high agreement with treatment decisions.
• Functional outcomes improved across treatment and complication subgroups.
• Complication rates were similar after surgical and conservative treatment.

## Graphical abstract

**Figure:**
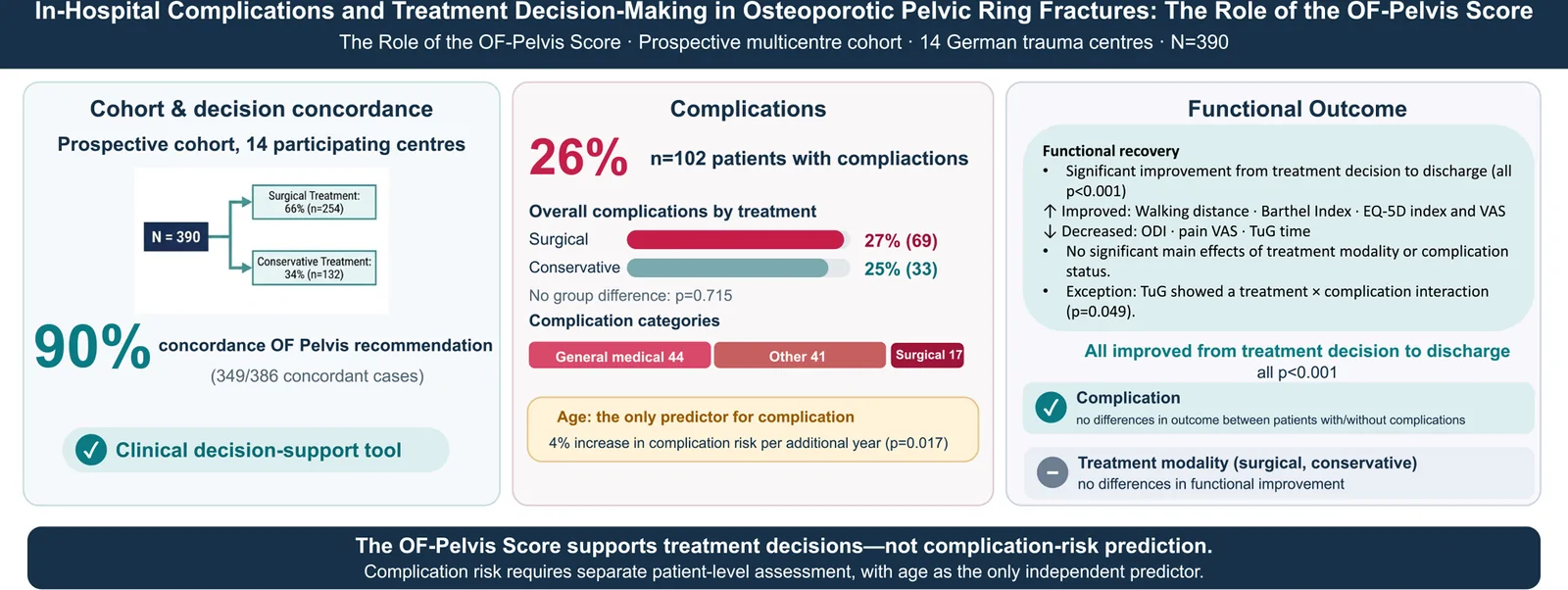

## 1 Introduction

Osteoporotic pelvic ring fractures (OPRF) represent a growing clinical challenge in geriatric trauma care (Rupp et al., 2021; Osche et al., 2025; Hu et al., 2023). Unlike high-energy trauma, these fractures typically occur after low-energy mechanisms in frail, elderly patients (Jehu et al., 2021) with compromised bone quality and comorbidities (Ullrich et al., 2021; Rommens et al., 2021). They are associated with prolonged immobilization, high in-hospital complication rates, and significant functional decline (Rommens et al., 2021).

In recent years, the development of the OF-Pelvis classification and its accompanying score (Ullrich et al., 2021; Spiegl et al., 2024) has provided clinicians with a structured framework to guide treatment decisions. Developed by the Working Group for Osteoporotic Fractures of the Spine Section of the German Society for Orthopaedics and Trauma (DGOU) the system incorporates both fracture morphology and patient-specific clinical parameters, including pain, mobility, neurological status, and comorbidity scores (Ullrich et al., 2021; Spiegl et al., 2024).

Although the OF-Pelvis Score correlates well with real-world treatment decisions and provides a functional basis for classifying fragility fractures (Spiegl et al., 2024), the correlation between this score and the incidence of complications during the hospital stay has not been thoroughly investigated. However, complications such as urinary tract infections, impaired wound healing, and prolonged immobility are common in this patient population and can significantly impact outcomes, especially for those who undergo surgery. (Rommens et al., 2021; Desmet et al., 2024; Wilson et al., 2021).

Despite these risks, it remains unclear to what extent fracture morphology, patient characteristics, or therapy modality independently impact adverse events. Several studies suggest that age and frailty, rather than the treatment itself, are stronger determinants of postoperative complications (Desmet et al., 2024; Forssten et al., 2023; Lo et al., 2024). The ability to identify high-risk patients early is critical, not only for tailoring treatment but also for optimizing resource allocation and rehabilitation planning.

This multicenter prospective study aims to contribute to closing this knowledge gap by evaluating the frequency, nature, and predictors of complications in patients with OPRF. Using the OF-Pelvis Score as a clinical stratification tool, we investigate whether treatment decisions were consistent with the score's recommendation and how these relate to complication rates and functional recovery, thereby assessing its usefulness as both a therapeutic and prognostic tool.

## 2 Materials and methods

Between 2021 and 2023, patients with pelvic fractures were enrolled, treated, and analysed as part of this prospective multicenter study. **Consecutive patients treated operatively or non-operatively for an isolated osteoporotic pelvic ring fracture at one of the participating centers were eligible for inclusion. Patients with pelvic ring fractures associated with primary bone tumors, metastatic disease, high-energy trauma, or concomitant injuries were not included in the study.** Data were collected from 14 participating centers and consolidated at the lead coordinating center. Ethical approval was granted by the respective institutional review boards at each participating site. Data were collected at the time of hospital admission, on the day of treatment decision, and at discharge. The day treatment decision (TD) was defined as the day on which the therapeutic strategy for managing the pelvic fracture was determined. In addition to demographic data and bone quality (Hounsfield Units, DEXA T-score, and quantitative CT), fracture morphology was classified according to the OF-Pelvis classification (OFP classification**,** Fig. 1). Additionally, the OF-Pelvis Score was calculated. The OF-Pelvis Score (Fig. 2) is a composite score that includes fracture morphology, patient mobility, pain, and neurological deficits attributable to the fracture. Patient health status is also incorporated, including ASA classification, the modified Frailty Index (mFI), and anticoagulant use. Additional modifiers such as fractures of the L5 transverse processes, fracture displacement, and bone marrow edema are also considered. A total OF-Pelvis Score of less than 8 suggests conservative treatment. A score greater than 8 indicates a recommendation for surgical treatment. A score of exactly 8 is considered a relative indication for surgery. The OF-Pelvis Score serves as a clinical decision support tool. The final treatment decision was made by the attending physician in accordance with best clinical practice.

**Fig. 1:**
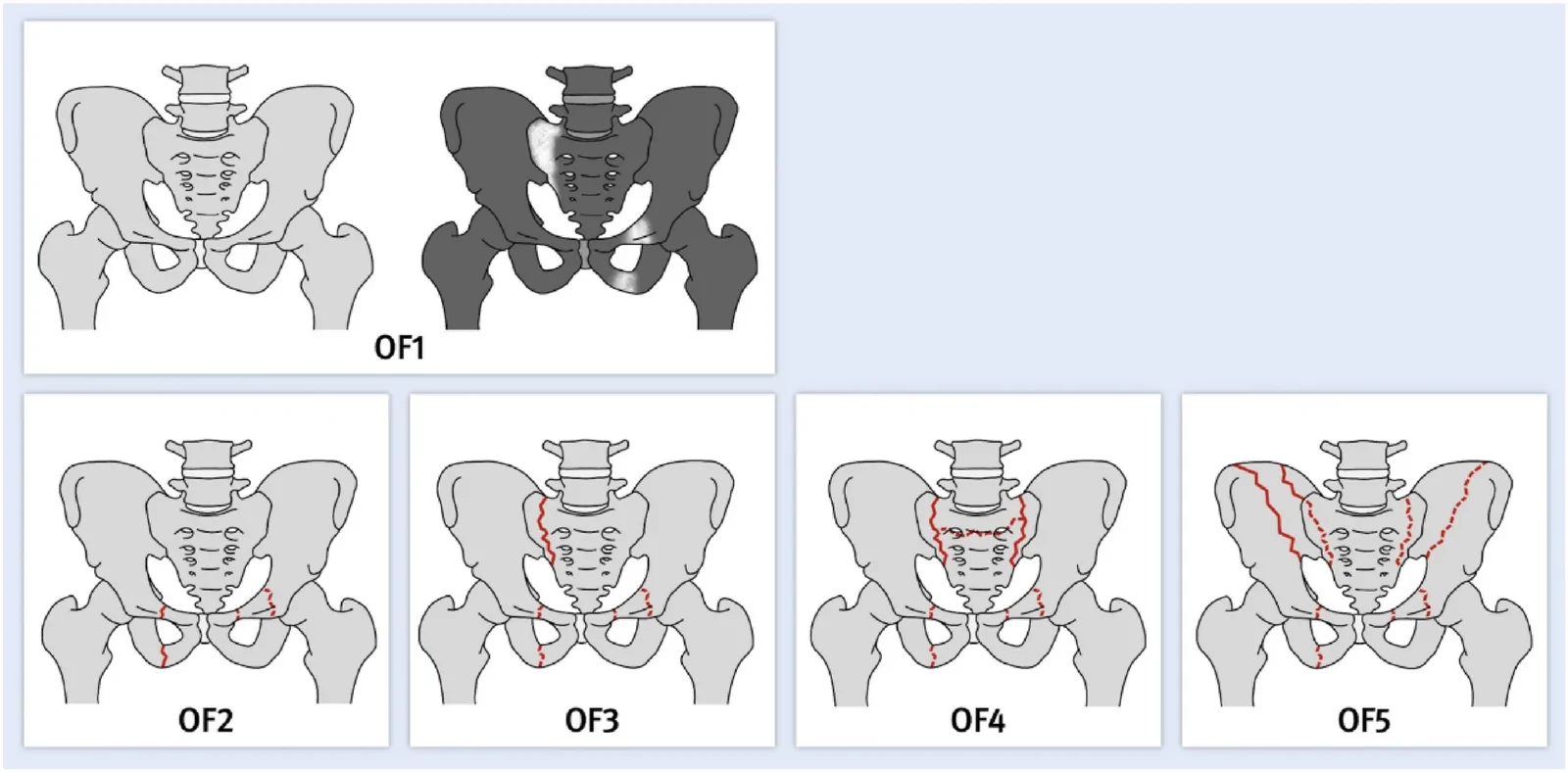
The OFP classification comprises five categories (Spiegl et al., 2026): OFP 1 is characterized by bone marrow edema at one or multiple sites of the pelvic ring without a fracture detectable on conventional CT. OFP 2 describes unilateral or bilateral fractures of the anterior pelvic ring. OFP 3 includes unilateral sacral fractures, either isolated or combined with anterior pelvic ring involvement. OFP 4 comprises bilateral sacral fractures, including H- and U-shaped configurations, with or without an associated anterior ring fracture. OFP 5 refers to iliac or transiliosacral fractures, which may likewise be accompanied by an anterior pelvic ring fracture. In the schematic representation, solid lines indicate characteristic fracture patterns within each category, whereas dashed lines denote optional additional fracture sites. MRI or dual-energy CT is required to identify bone marrow edema.

**Fig. 2:**
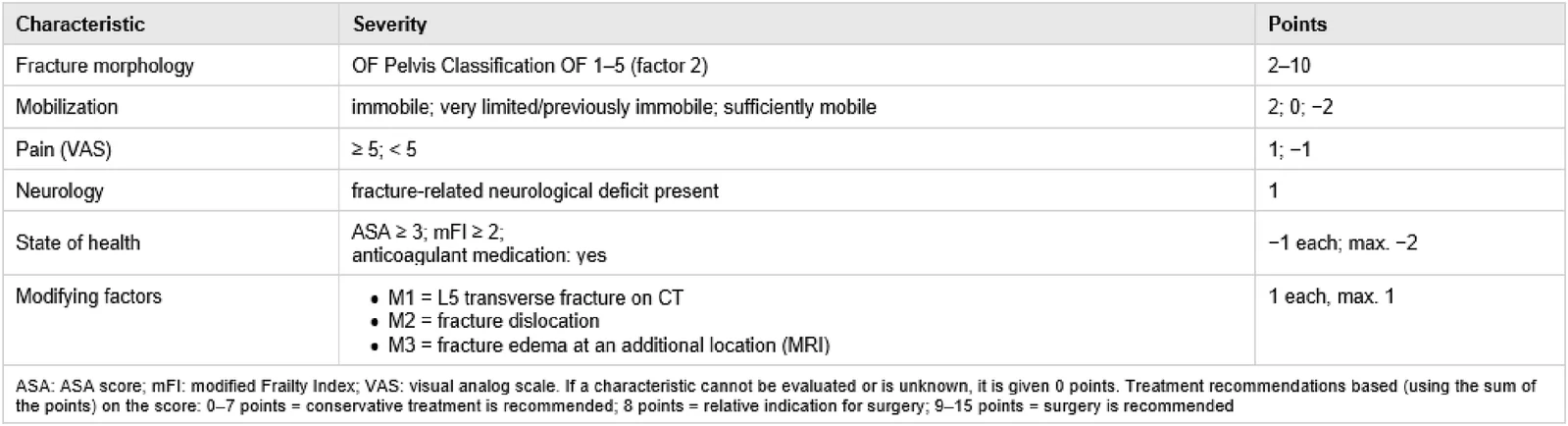
OF-Pelvis Score combining fracture morphology and patient-related factors to guide treatment: 0–7 points indicate conservative treatment, 8 points a relative indication for surgery, and 9–15 points surgical treatment (Spiegl et al., 2025).

The treatment method, surgical or conservative, was recorded. Functional outcomes were assessed based on patient mobility, the Oswestry Disability Index (ODI), Barthel Index, EQ5D-5L Index Score and EQ5D Visual Analogue Scale (EQ5D-VAS), Timed Up and Go (TuG) test, pain assessed using a visual analogue scale (VAS), and the patient's self-reported walking distance before treatment and at discharge. Mobility was documented as a binary variable, categorizing patients as either independently mobile or non-mobile. Non-mobile patients included those who were bedridden or wheelchair-bound.

The following complications during hospitalization were recorded: urinary tract infection, pneumonia, pressure ulcers, pulmonary embolism, surgical site complications, wound healing disorders, bleeding, implant malposition, and need for revision surgery.

### 2.1 Statistics

Differences in age, bone quality, and length of hospital stay were assessed using a univariate general linear model (GLM). Treatment type and occurrence of complications were entered as fixed factors (binary variables). Differences in the distribution of binary variables (e.g., sex, mobility, presence of osteoporosis, neurological deficits) and categorical variables (e.g., ASA classification, OF-Pelvis classification) between treatment groups and in relation to complications were analysed using Chi-square or Fisher's exact tests. If the test shows significance the contingence coefficient will be given. Logistic regression was used to evaluate the influence of age, ASA classification, pain, mobility, and treatment approach on the risk of complications. To assess the impact of complications and differences in treatment modality on functional outcomes over time, a repeated-measures general linear model (GLMrm) was used. The functional outcome at treatment decision and discharge was used as within-factor (repeated measure). This analysis was performed separately for each functional outcome variable (walking distance, ODI, Barthel Index, TuG, EQ5D index value, and EQ5D-VAS, and pain VAS). To account multiple comparisons, post hoc pairwise tests were Bonferroni corrected. Mean and standard deviations were calculated for continuous variables, while frequencies and percentages were used for categorial variables. Results of the functional outcome analyses are presented graphically as means and corresponding 95% confidence intervals. As an additional sensitivity analysis, predefined general medical complications (urinary tract infection, pneumonia, pressure ulcer, and pulmonary embolism) were analysed separately from surgery-related complications (wound healing disorder, bleeding, implant malposition, and revision surgery). The occurrence of general medical complications was compared between surgical and conservative treatment groups using Chi-square or Fisher's exact tests, as appropriate. All statistical analyses were performed using SPSS version 29 (IBM Corp. Released, 2022. IBM SPSS Statistics for Windows, Version 29.0. Armonk, NY: IBM Corp). A p-value of <0.05 was considered statistically significant.

### 2.2 Ethics approval and consent to participate

The study was approved by the institutional review boards of all participating centers. All procedures were performed in accordance with relevant guidelines and regulations (Declaration of Helsinki). Written informed consent was obtained from all participants or their legal representatives prior to inclusion.

### Ethical approval

The study was approved by the local institutional ethics committees of all participating centers.

- University Hospital Leipzig: Ethikkommission der Medizinischen Fakultät: 522/20-ek
- Klinikum Traunstein: Bayerische Landesärztekammer: 2017-136
- BG Klinikum Bergmannstrost Halle: Ärztekammer Sachsen-Anhalt: 88/21
- Universitätsklinikum Jena: Ethikkommission: 5507-0418- Universitätsklinikum Schleswig-Holstein, Kiel: Medizinische Fakultät der CAU zu Kiel: AZ B213/21
- Sana Klinikum Borna: Sächsische Landesärztekammer: EK-BR-30/22-1
- Klinikum Nürnberg: Ethik-Kommission FAU Erlangen-Nürnberg: 309_17 Bc (For other centers, local IRB approvals were also obtained; reference numbers available on request.)

### 2.3 Consent to publish

The manuscript does not include any individual person's data in any form (images, videos, or identifiable information).

## 3 Results

Data from a total of 390 patients (57 men, 333 women) with a mean age of 80 ± 8 years (range: 54–99 years) were analysed. Treatment information was available for 386 patients; 254 patients (66%) underwent surgical treatment and 132 patients (34%) were treated conservatively. For four patients, information on treatment modality was unavailable. The most frequently observed fracture types were OFP3 (n = 173, 44%) followed by OFP4 (n = 164, 42%). OFP2 and OFP5 fractures were identified in 29 (7%) and 21 (5%) patients, respectively. OFP1 fractures were diagnosed in 3 patients (1%).

The mean time from fracture to hospital admission was 12 ± 24 days (range: 0–210 days). A total of 132 patients (34%) were treated conservatively, while 254 patients (66%) underwent surgical treatment. The average length of hospital stay was significantly longer in patients with complications (16 ± 10 days) compared to those without complications (11 ± 6 days, p < 0.001). Table 1 presents the patients characteristics stratified by treatment group and occurrence of complication.

**Table 1:** *Baseline characteristics of patients with osteoporotic pelvic ring fractures, stratified by treatment type (surgical, conservative) and complication status*. *Treatment modality was unavailable for four patients; therefore, treatment-specific subgroup analyses include 386 of the 390 patients.*

Patient Characteristics	Total (N = 390)	Surgery (N = 254)		p	Conservative (N = 132)		p
		Complication	No Complication		Complication	No Complication	
**Women/Men**	333/57	59/10	157/28	1.000	29/4	84/15	0.781
Age (years)	80 ± 8	82 ± 7	79 ± 8	0.008	83 ± 9	81 ± 9	0.161
Hospitalization [d]	13 ± 8	18 ± 10	13 ± 6	<0.001	13 ± 9	9 ± 5	0.002

Bone Quality							
HU	29 ± 45	28 ± 45	34 ± 50	0.347	14 ± 26	26 ± 37	0.114
DEXA	−0,24 ± 0,93	−0.33 ± 1.09	−0.15 ± 0.70	0.138	−0.22 ± 0.86	−0.37 ± 1.18	0.517
QCT	−0,2 ± 0,8	−0.2 ± 0.9	−0.2 ± 0.9	0.972	0 ± 0	−0.1 ± 0.6	0.424

ASA classification				0.469			0.197
I	6 (2%)	0 (0%)	3 (2%)		0 (0%)	3 (3%)	
II	145 (37%)	22 (32%)	72 (40%)		9 (29%)	41 (43%)	
III	216 (55%)	44 (64%)	101 (56%)		19 (61%)	49 (51%)	
IV	14 (4%)	3 (4%)	5 (3%)		3 (10%)	3 (3%)	
Unknown	9 (2%)						

Osteoporosis				1.000			1.000
Yes	377 (97%)	66 (96%)	176 (95%)		33 (100%)	98 (99%)	
None	13 (3%)	3 (4%)	9 (5%)		0 (0%)	1 (1%)	

Neurological Deficit				0.733			0.573
Yes	13 (3%)	2 (3%)	8 (4%)		0 (0%)	3 (3%)	
None	377 (97%)	67 (97%)	177 (96%)		33 (100%)	96 (97%)	

OF-Pelvis classification				0.002			0.970
OFP1	3 (1%)	1 (1%)	0 (0%)		0 (0%)	2 (2%)	
OFP2	29 (7%)	0 (0%)	3 (2%)		6 (18%)	20 (20%)	
OFP3	173 (44%)	24 (35%)	67 (36%)		20 (61%)	60 (61%)	
OFP4	164 (42%)	33 (48%)	109 (59%)		6 (18%)	14 (14%)	
OFP5	21 (5%)	11 (16%)	6 (3%)		1 (3%)	3 (3%)	

OF-Pelvis Score	9 ± 2	10 ± 2	10 ± 2	0.142	7 ± 2	7 ± 2	0.559

Mobile				0.777			0.054
Yes	218 (56%)	28 (41%)	79 (43%)		24 (73%)	87 (88%)	
No	172 (44%)	41 (59%)	106 (57%)		9 (27%)	12 (12%)	

Complications occurred in 102 patients (26%), with no significant association with sex (14 men [25%], 88 women [27%], p = 0.871). Complications occurred in 69 (27%) of patients who underwent surgery and in 33 (25%) of patients who received conservative treatment (p=0.715). Among patients with available treatment information, 349 of 386 (90%) were treated in accordance with the recommendation of the OF-Pelvis Score. Compliance of the OF-Pelvis Score recommendation showed no significant association on complication rates (p = 0.139). ASA classification among patients ranged from ASA I (n = 6) to ASA IV (n = 14). Most patients were classified as ASA III (n = 216, 55%), followed by ASA II (n = 145, 37%). No significant association between ASA classification and occurrence of complications could be demonstrated (p = 0.074). Fracture morphology according to the OFP classification showed a significant association with the occurrence of complications (contingency coefficient = 0.168, p = 0.029). This association was not significant in conservatively treated patients (p = 0.970), but it was significant in surgically treated patients (contingency coefficient = 0.251, p = 0.002). An overview of the complications identified in patients with pelvic fractures is provided in Table 2. General complications occurred in 44 patients: 25 (10%) among those treated surgically and 19 (14%) among those treated conservatively (p=0.182). Additional complications specific to surgery were observed in 17 (7%) of the 254 patients who underwent surgery.

**Table 2:** Identified complications for the complete group of patients with pelvic fractures and for the surgically and conservatively treated patients.

	Total	Surgery	Conservative	P
Urinary tract infection	34	20	14	0.449
Pneumonia	5	2	3	0.343
Pressure ulcer	4	3	1	1.000
Thrombosis	-	-	-	-
Pulmonary embolism	1	-	1	0.342
Surgical site complication				
Wound healing disorder	2	2	-	
Bleeding	4	4	-	
Implant malposition	3	3	-	
Revision surgery	8	8	-	
Other complications	41	27	14	0.994

Logistic regression was performed using data from 377 patients to predict complications, 13 patients were excluded due to missing information in at least one of the predictor variables. The model achieved a prediction accuracy of 74.5%, with a Nagelkerke R^2^ of 0.06, indicating poor to moderate explanatory power. Of 100 patients who actually experienced complications, the model correctly predicted complications in only 4 cases (4%). In 96 patients, complications occurred but were not predicted by the model. All patients without complications were correctly classified as such. In total, the model predicted no complications for 373 patients, of whom 277 (74.3%) were correctly classified.

Within the logistic regression model, age was the only variable independently associated with complication occurrence. For each additional year of age, the odds of complication increased by approximately 4% (OR 1.04, 95% CI 1.01–1.07, p = 0.017). No significant associations were found for the other variables: ASA classification (p > 0.397; ASA I as reference), pain (p = 0.952), mobility (p = 0.504), or treatment type (p = 0.616).

The repeated-measures general linear model (GLM) demonstrated statistically significant improvements in all functional outcome measures (EQ5D, walking distance, ODI, Barthel Index, TuG and pain) during the inpatient period (all p < 0.001). No significant main effects of complications or treatment type were observed for these outcomes, except for the TuG, where an interaction effect between complication status and treatment type was detected (p = 0.049).

Post hoc pairwise comparisons revealed that, at the time of treatment decision (TD), surgically treated patients who later developed complications required on average 20 s longer to complete the TuG compared to conservatively treated patients (p = 0.038). Among patients without complications, no significant difference in TuG times at TD was observed between treatment groups. The TuG test was completed by 148 patients at TD and by 292 patients at the time of discharge (p < 0.001). The results of these functional outcome variables over time are illustrated in Fig. 3.

**Fig. 3:**
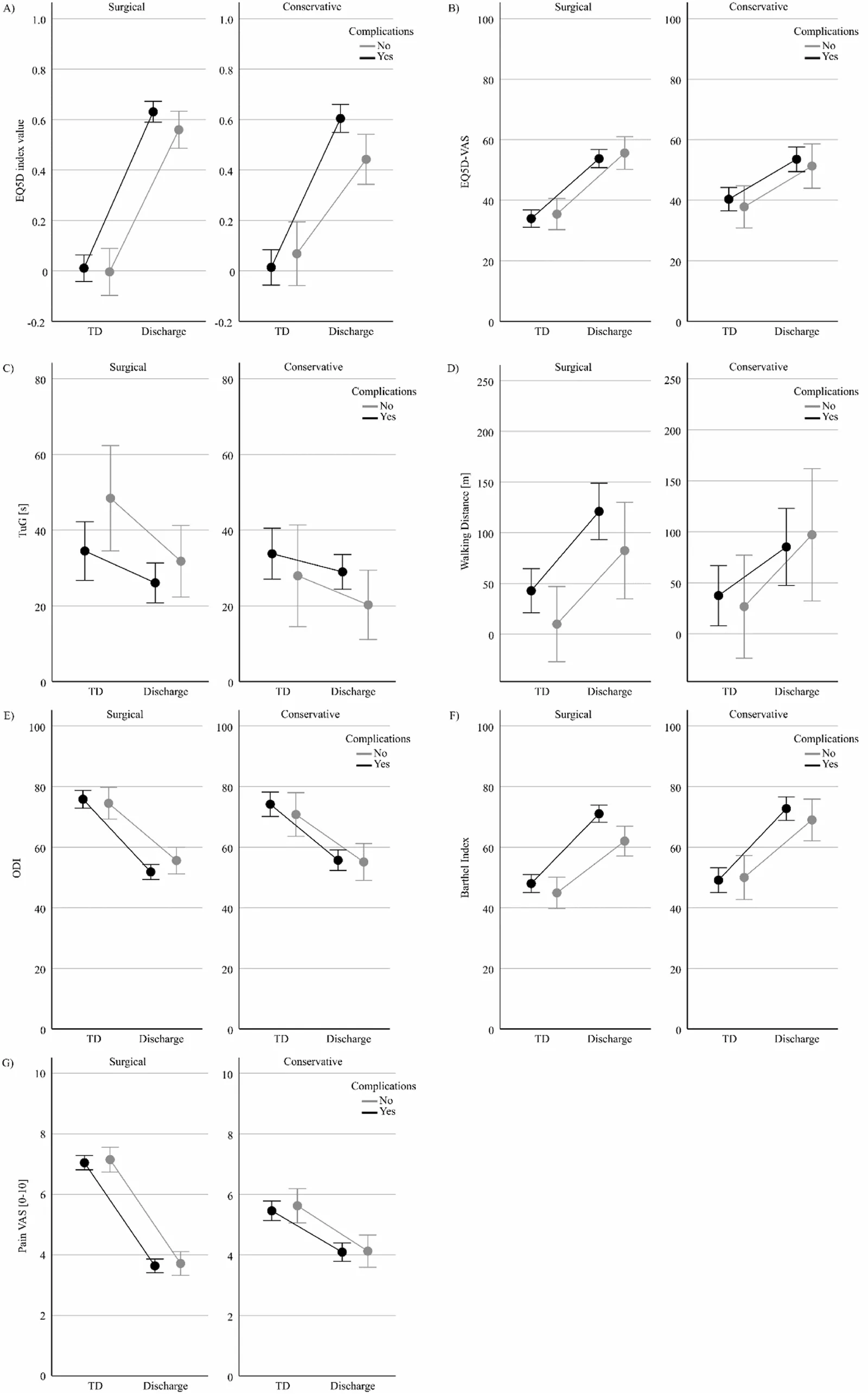
Functional outcome of patients with osteoporotic pelvic fractures at the day of treatment decision (TD) and at discharge. Results are presented separately for surgically and conservatively treated patients, and further differentiated by the occurrence of complications. Values are shown as means with 95% confidence intervals.

## 4 Discussion

This prospective multicenter study documented 102 in-hospital complications (26%) in patients with osteoporotic pelvic ring fractures (OPRFs), most commonly urinary tract infections, wound-related problems, hemorrhage, and revision surgeries. Overall complications were not more frequent in surgically treated patients than in those treated conservatively. The overall in-hospital complication rate in our cohort (26%) was comparable to the 27.9% reported by Rommens et al. (2021)**.** In contrast to their findings, however, complication rates in our cohort did not differ significantly between surgically and conservatively treated patients (27% vs. 25%; p=0.715). Consistently, multivariable logistic regression showed that treatment modality was not independently associated with complication occurrence (p=0.616). Patients’ age was the only variable independently associated with complications. Each additional year of age was associated with a 4% increase in the odds of experiencing a complication. This may reflect the accumulation of comorbidities with age, which were not fully captured in our dataset but may have contributed to vulnerability. This finding aligns with previous research that also identified advanced age as a major contributing factor to negative outcomes in patients with fragility fractures (Forssten et al., 2023; Lo et al., 2024; Höch et al., 2017; Bhattacharya et al., 2016; Hill et al., 2001; Keppler et al., 2024).

Neither the OF-Pelvis Score, ASA classification, baseline pain, nor initial mobility were independently associated with complication risk. The OF-Pelvis fracture classification showed a significant association with complications in surgically treated patients. This underlines the importance of not only the treatment decision itself, but also the underlying injury pattern and its interaction with patient-related risk factors. While our study focused on the OF system, prior research using the Fragility Fracture of the Pelvis (FFP) classification has demonstrated that more unstable morphologies (FFP III and IV) are linked to increased complications (Rommens et al., 2021). Combining morphological and systemic risk assessments may thus provide a more complete prognostic framework.

In contrast, ASA classification, baseline pain levels, initial mobility, and the OF-Pelvis Score were not independently associated with in-hospital complications. These findings suggest that patient-related factors, particularly age, may be more relevant to complication occurrence than treatment modality alone (Forssten et al., 2023; Lo et al., 2024; Höch et al., 2017; Rogers et al., 2020; Krappinger et al., 2010). Supporting this view, Keppler et al. showed that orthogeriatric co-management in pelvic and acetabular fractures reduces complications such as urinary tract infections and revision surgeries and enables earlier mobilization, highlighting the value of standardized interdisciplinary protocols addressing frailty and comorbidity more effectively than surgical decision-making alone (Keppler et al., 2024). Consistently, Forssten et al. identified frailty, measured via the Orthopedic Frailty Score, as a key independent predictor of mortality and complications in over 66,000 geriatric pelvic fracture patients (Forssten et al., 2023). Rege et al. extended this evidence to younger trauma patients, demonstrating that frailty, measured by a modified frailty index (mFI), was a stronger predictor of mortality and severe complications than age or ASA classification, underlining the need to assess frailty across all age groups (Rege et al., 2018). These findings support the importance of patient-related vulnerability in addition to fracture morphology and treatment modality when considering complication risk and highlight the potential value of integrating frailty assessment into the management of osteoporotic pelvic ring fractures.

A detailed analysis of complications revealed that the majority were non-life-threatening but clinically significant events. Urinary tract infections (34 cases) and wound-related issues, including hematoma and material malposition were among the most impactful. Although rare, more serious events like pulmonary embolism and pneumonia were also observed. These rates are within the range reported in similar large-scale cohort studies (Rommens et al., 2020, 2021, 2022; Höch et al., 2017) and confirm the need for meticulous perioperative monitoring, especially in surgically treated elderly patients (Rommens et al., 2019, 2021; Wilson et al., 2021; Höch et al., 2017).

Functional outcomes improved significantly across all measures, regardless of treatment group or complication status, supporting the value of structured multidisciplinary care. This aligns with prior research showing that rehabilitation protocols, not treatment type alone, drive recovery (Krappinger et al., 2010; Sivapathasuntharam et al., 2022; Osterhoff et al., 2019; Saito et al., 2022). Nevertheless, a closer look reveals that the TuG performance was significantly delayed in patients who developed complications and underwent surgical treatment. This interaction effect suggests that while complications may not dictate outcomes, they can hinder short-term functional recovery. These results are consistent with others indicating that, while postoperative complications hinder recovery of mobility, they do not prevent long-term functional outcomes. Conversely, patients treated for OPRFs had lower mortality rates in the long-term follow-up (Höch et al., 2017; Osterhoff et al., 2019). However, a limitation is that patients who were not mobile or unable to perform the TuG were excluded from analysis, which may have introduced selection bias.

The EQ5D index value and EQ5D-VAS measures showed subjective health improvements across all patient subgroups. Pain scores declined most notably in the surgical group, which aligns with previous data showing that mechanical stabilization reduces nociceptive stimuli in unstable pelvic injuries (Barei et al., 2010). These pain reductions also likely facilitated faster participation in rehabilitation activities and thus other outcome measures.

Despite no differences in complication rates among surgically treated patients compared to conservatively treated patients, this group demonstrated the greatest pain reduction and fastest subjective recovery. This emphasizes that, while relevant, complications must be considered in the context of therapeutic benefit. Not all complications carry the same clinical significance, and their occurrence does not negate the benefits of surgical stabilization when indicated.

Comparative studies repeatedly point to variability in the treatment of fragility fractures across institutions (Osche et al., 2025). This supports the idea of introducing standardized instruments, such as the OF-Pelvis Score, to improve the quality of care and reporting of outcomes.

The results of this study therefore reflect the reality of healthcare provision in the German-speaking region. Building on prior work the study confirms the clinical concordance of the OF-Pelvis Score with actual treatment decisions (90% conformity) and highlights its dynamic application for guiding individualized therapy. Among the 390 patients evaluated, a majority had OFP3 and OFP4 fractures, reflecting the distribution commonly reported in similar cohorts (Rommens et al., 2022). Surgical treatment was more common among patients with OFP4 and OFP5 fractures, which supports the view that unstable morphologies require operative stabilization (Rommens and Hofmann, 2013).

Limitations of the current study include the heterogeneous implementation across 14 participating centers and thus potential variations in surgical technique, and incomplete datasets for some variables, limiting the power of regression models. Despite these, the study provides a robust real-world snapshot of current practices in the treatment of osteoporotic pelvic fractures in Germany. The functional outcome measures are standard in the evaluation of these patients, although some measures, particularly the TuG test, may have introduced selection bias because they could not be performed in all patients.

In this large prospective multicenter study, in-hospital complications occurred in one out of four patients with osteoporotic pelvic ring fractures, with increasing age being independently associated with complication risk. Surgical treatment itself was not independently associated with complications. Instead, age emerged as the sole significant patient-related factor. The OF-Pelvis Score demonstrated high agreement with real-world treatment decisions but did not predict complication risk. Despite the frequency of complications, functional outcomes improved across all subgroups, highlighting the resilience of structured multidisciplinary care. These findings underscore the importance of considering patient-related factors, particularly age, when assessing complication risk.

## Ethics declaration

Written informed consent to take part in the study and to publish the article has been obtained from all participants or their legal representatives. The privacy rights of participants have been observed.

This study was performed in compliance with relevant laws, regulatory frameworks and guidelines where the research took place. The study was approved by the institutional ethics committees of the participating centers, including the Ethics Committee of Universitätsklinikum Jena (Approval No. 5507-0418).

## Data availability

The datasets generated and analysed during the current study are available from the corresponding author on reasonable request. De-identified data, the statistical analysis plan and codebook will be provided upon request.

## Author contributions

F.C.K. and P.S. contributed equally.

Felix C. Kohler: Conceptualization, Methodology, Investigation, Data curation, Formal analysis, Writing – original draft, Writing – review & editing, Project administration.

Philipp Schenk: Conceptualization, Methodology, Investigation, Data curation, Formal analysis, Writing – original draft, Writing – review & editing.

Pamela Schanderl: Investigation, Data curation, Writing – review & editing.

Volker Zimmermann: Investigation, Data curation, Writing – review & editing.

Bernhard Ullrich: Conceptualization, Methodology, Investigation, Data curation, Writing – review & editing.

Martin Naisan: Investigation, Data curation, Writing – review & editing.

Erik Wegner: Investigation, Data curation, Writing – review & editing.

Michael Müller: Investigation, Data curation, Writing – review & editing.

Frank Hartmann: Investigation, Data curation, Writing – review & editing.

Klaus J. Schnake: Conceptualization, Methodology, Supervision, Writing – review & editing.

Imke Schmerwitz: Investigation, Data curation, Writing – review & editing.

Lars Behr: Investigation, Data curation, Writing – review & editing.

Max J. Scheyerer: Investigation, Data curation, Writing – review & editing.

Robert Pätzold: Investigation, Data curation, Writing – review & editing.

Sebastian Grüninger: Investigation, Data curation, Writing – review & editing.

Georg Osterhoff: Conceptualization, Methodology, Supervision, Writing – review & editing.

Ulrich J. A. Spiegl: Conceptualization, Methodology, Supervision, Project administration, Writing – review & editing.

All authors approved the final version and agree to be accountable for all aspects of the work.

## Use of large language models

During manuscript preparation, large language models (e.g., ChatGPT) were used only to assist with language editing. The authors reviewed and edited the content as needed and take full responsibility for the manuscript's content.

## Funding

This research received no specific grant from any funding agency in the public, commercial or not-for-profit sectors.

## Declaration of competing interests

The authors declare that they have no known competing financial interests or personal relationships that could have appeared to influence the work reported in this paper.
